# Inflammatory Myofibroblastic Tumor: A Rare Tumor in the Tongue

**DOI:** 10.1155/2013/787824

**Published:** 2013-03-27

**Authors:** Nur Yucel Ekici, Tuba Bayindir, Ahmet Kizilay, Nasuhi Engin Aydin

**Affiliations:** ^1^Department of Otorhinolaryngology, Kozan State Hospital, Adana, Turkey; ^2^Department of Otorhinolaryngology, Faculty of Medicine, Inonu University, Malatya, Turkey; ^3^Department of Pathology, Faculty of Medicine, Inonu University, Malatya, Turkey

## Abstract

Inflammatory myofibroblastic tumor is composed of myofibroblast and inflammatory cell infiltration of the tissue. Malign transformation and recurrence rate of this tumor is rare and accepted as benign fibroinflammatory disease. The main etiology is unclear, but infection, trauma, and immunologic event are accused. In this study, we presented a 75-year-old man with a mass on his tongue, which was diagnosed as “inflammatory myofibroblastic tumor.” This type of tumor is rarely seen in the tongue and might be difficult to diagnose. Complete mass excision was provided for an adaquete treatment.

## 1. Introduction

Inflammatory myofibroblastic tumor (IMT) is a rare mesenchymal tumor [1]. The etiology of IMT is contentious, also the nature of this tumor type (benign or malign) is still controversial [1–3]. But, because of its rarity, the current conception is altered from a benign reactive process to an intermediate neoplasm [4]. Different terms such as; inflammatory pseudotumor, histiocytoma, plasma cell histiocytoma complex, plasma cell granuloma, fibrohistiocytoma, xanthomatous granuloma, myxoid hamartoma, xanthomatous pseudotumor, spindle cell pseudotumor, inflammatory fibrosarcoma, benign myofibroblastoma, and inflammatory myofibroblastic proliferation were used for defining this tumor. World Health Organization (WHO) had consolidated this term as IMT in 1994 and declared it as a diagnostic classification for intermediate soft-tissue myofibroblastic neoplasm according to its well reproducible histological morphology [4, 5].

The tumor reported in the lung originally, but extra-pulmonary form, has been described in different organs and anatomic sites [6, 7]. Regardless of the site of origin, it is a circumscribed mass-forming process, composed of myofibroblasts, fibroblasts, histiocytes, and plasma cells. Although stromal fibrosis is a finding that is usually seen, tumor tends to be limited in the focal area [6, 8]. IMT is preferentially occurring in children and young adults [6]. In this report, we presented a 75-year-old male patient with inflammatory myofibroblastic tumor of the tongue presenting with pediculated large mass.

## 2.   Case Report

**Figure 1:**
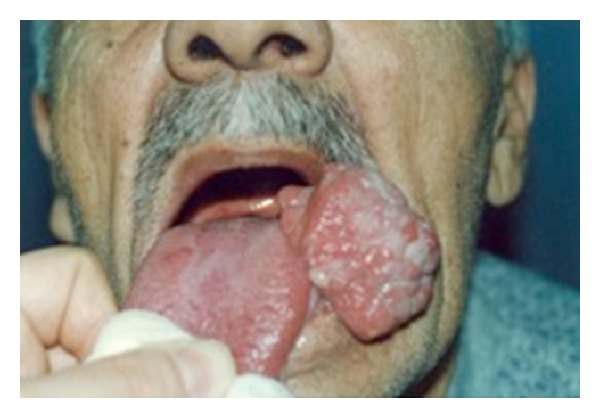
The mass on the left side of the tongue.

A 75-year-old man was referred to our clinic with a painless, pediculated large mass on the left side of the tongue. The lesion grew rapidly within four months. Oral examination revealed an approximately 4 centimeter (cm) in diameter, hard, partially ulcerated, and pediculated large mass on the left side of the tongue (Figure 1). There was no regional lymphadenopathy, and the remainder of physical examination was reported to be normal. The patient was not defined repeated injury and weight loss within the last year. The patient uses tobacco, one pocket per day since 45 years. In his medical history, advanced coronary artery disease and bronchial asthma were noted. 

**Figure 2:**
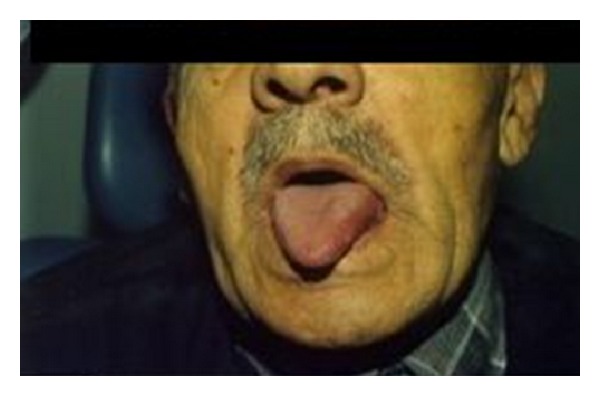
View of the tongue after total surgical excision.

Initially, the incisional biopsy was done from mass, and the histopathological examination showed that the lesion was either a spindle cell carcinoma with an unusual appearance or an inflammatory myofibroblastic tumor. Because the diagnosis was uncertain, the lesion was excised totally with a 1 cm of surrounding healthy tissue (Figure 2). 

## 3. Histopathological Examination

**Figure 3:**
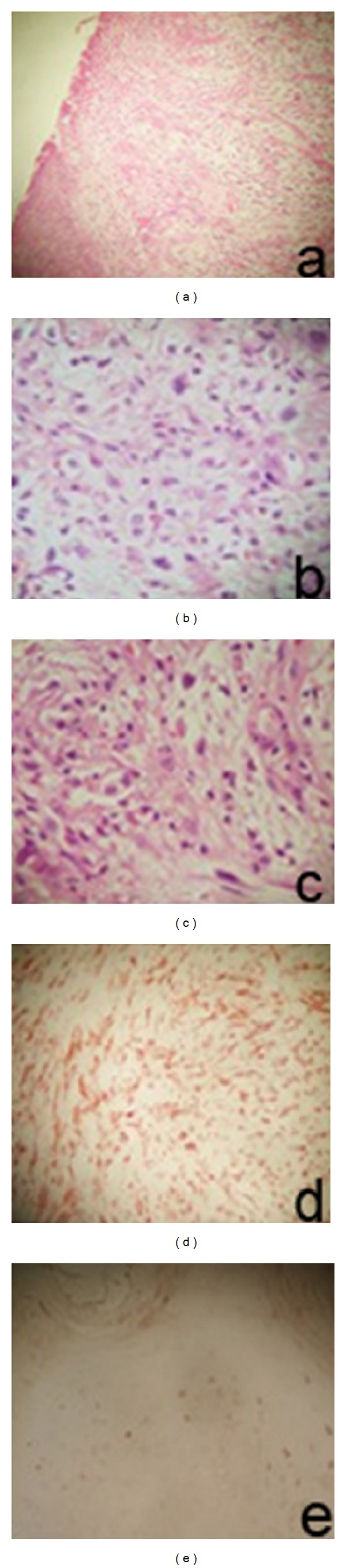
(a) Granulation tissue-like view with mucosal ulceration (H.E. ×100). ((b),(c)) Spindle-shaped structure pleomorphic cellular elements in granulation tissue (H.E. ×200). (d) Positivity of vimentin that shows mesenchymal phenotype of cells (immunohistochemistry, ×200). (e) HPV antigen positivity on the adjacent mucosa squamous epithelium (immunohistochemistry, ×200).

The histopathologic examination showed atypical inflammatory myofibroblastic tumor with large mucosal ulceration, and the surgical margins were tumor-free. On immunohistochemical examination, less keratinocyte positivity with human papilloma virus (HPV) was showed. Most spindle cells were strongly and diffusely stained positive with vimentin and alpha-smooth muscle actin, whereas negative with S-100 protein (Figures 3(a)–3(e)). Because of vimentin ve smooth muscle actin positivity, the final diagnosis was IMT. 

The patient died one year later due to cardiological problems. During followup period, there was no recurrence on surgery region.

## 4. Discussion

IMT may occur at nearly every site of the body, including lung, hypopharynx, central nervous system, abdomen, orbit, extremities, nose and paranasal sinuses, and oral cavity [3, 7, 9–11]. These tumors are rarely encountered in the tongue.

The etiology of this tumor is still unclear, but infection, trauma, or abnormal immunological reactions are accused. Also there are some reports that present Epstein-Barr virus [12] or herpes simplex type II [13] as the etiologic factor for IMT. Recent cytogenetic and molecular observations have shown abnormalities in chromosomal band 2p23, resulting in a rearrangement of the ALK gene [10]. In our case, our patient did not experience any trauma in his history. On immunohistochemical examination, less keratinocyte positivity with human papilloma virus (HPV) was showed, but the exact etiological factor was not determined. 

The tumor has a locally aggressive nature and may grow slowly or rapidly. Usually the patients apply with progressive symptoms, which depend on the mass effect. Though the pathogenesis and etiology of the IMT has not been clearly understood, it has been suggested that the host defence mechanisms against the infectious agents, microorganisms, neoplasms, foreign bodies, and traumas may play a role in the etiology of this type of tumor [12–14]. 

Diagnosis of IMT is done by immunohistochemical evaluation. Also the histopathologic diagnosis of this type of tumor is difficult. This type of tumor is dominated by proliferating myofibroblastic cellular population in a background of plasma cells, lymphocytes, eosinophils, and blood vessels. Significant stromal fibrosis and a tendency to the focal limitation can be seen in most of the cases [6]. Spindle cells were reacted for smooth-muscle actin and vimentin. Histopathologically, this type of tumor is defined as benign-natured, well-defined, and unencapsulated tumor. But, because of the locally aggressive behavior and tendency to recurrence, the differential diagnosis from malignant tumors has been done. On histopathological examination, on the cross section view, the lesion can be seen as a scar-like soft tissue, that contains multifocal lypmhocyte, histiocyte, plasma cells, and eosinophiles, without hemorrhagic and necrotic areas. Also, spindle cells with eosinophilic cytoplasm and oval-elongated shaped nucleus can be seen between the inflammatory cells. These cells stain positive with vimentin and smooth muscle actin, whereas negative with S-100 and desmin. The lymphoid cells are CD20 positive B cells and CD3 positive T cells [6]. A tumor with spindle cell proliferation in a fibroinflammatory background and myofibroblastic differentiation on immunocytochemistry is regarded as an IMT [14]. The final diagnosis was, therefore, thought to that of an inflammatory myofibroblastic tumor of the tongue. In our case, histopathologic examination showed atypical inflammatory myofibroblastic tumor with large mucosal ulceration. On immunohistochemical examination, most spindle cells were strongly and diffusely stained for vimentin and alpha-smooth muscle actin and showed less keratinocyte positivity with human papilloma virus (HPV) (Figures 3(a)–3(e)). 

 This type of tumor is rarely seen in the tongue and might be difficult to diagnose. Differential diagnosis of IMT in the tongue included some foreign body reactions, myoepiteliomas, fibrosarcoma, and solitary fibrous tumors [11, 15, 16]. Foreign body reactions represent a fibrohistiocytic inflammatory process and granulocyte infiltration [16], but we could not find specific foreign material. IMT can be restricted to fibrohistiocytic or hyalinizing spindle cell lesions with an inflammatory appearance in the tongue. Myoepiteliomas with deposits of amorphous hyaline material can be differentiated by identifying the ductal structures, plasmacytoid cells, and immunoreactivity for keratin [15]. Spindle cell morphology, smooth-muscle cells, and matrix collagen present both in fibrosarcoma and IMT. Myofibroblasts found in inflammatory myofibroblastic tumors, however, stain for alpa-smooth muscle actin, fibronectin, and vimentin, but not for desmin and caldesmon. This distinguishes these lesions from fibrosarcoma, which should stain for both desmin and caldesmon [11]. Low-grade myofibroblastic sarcomas have been reported in the tongue [17], but their diffuse infiltrative growth, nuclear atypia, and immunoreactivity for desmin distinguish these lesions from IMT. The diagnosis of IMT was established as this recurrent tongue mass did not show any malignant phenotype. In a report, it has been shown that in various case reports, IMT of the oral cavity and head and neck region have been described priorly as benign lesions. Therefore, it has been suggested that this lesions should be treated as a low-grade mesenchymal neoplasm [6]. 

Because of the confusion in management of IMT, different treatment modalities, such as steroid therapy, excision biopsy, curettage with wide excision, radical surgery, radiotherapy, and chemotherapy can be offered [6, 10, 14]. However, reports of sarcomatous conversion, infiltrative growth, and local recurrence warrant adequate local treatment [14]. Up-to-date, recurrences were reported in nonoral and nonhead and neck sites [10]. In our case, the lesion was excised with 1 cm surrounding healthy tissue; and during followup period, no recurrence was seen in the surgery region.

In conclusion, IMT is a rare tumor type of the tongue, and the diagnosis of the tumor or the differential diagnosis from cancer may be difficult. But, IMT may be kept in mind in the differential diagnosis of the tongue masses.
